# Melatonin Modulation of Radiation and Chemotherapeutics-induced Changes on Differentiation of Breast Fibroblasts

**DOI:** 10.3390/ijms20163935

**Published:** 2019-08-13

**Authors:** Alicia González-González, Enrique García Nieto, Alicia González, Cristina Sánchez-Fernández, Carolina Alonso-González, Javier Menéndez-Menéndez, José Gómez-Arozamena, Samuel Cos, Carlos Martínez-Campa

**Affiliations:** 1 Department of Physiology and Pharmacology, School of Medicine, University of Cantabria and Instituto de Investigación Sanitaria Valdecilla (IDIVAL), 39011 Santander, Spain; 2 Department of Anatomy and Cellular Biology, School of Medicine, University of Cantabria, 39011 Santander, Spain; 3 Department of Medical Physics, School of Medicine, University of Cantabria, 39011 Santander, Spain

**Keywords:** melatonin, breast cancer, aromatase, adipocyte, differentiation

## Abstract

Melatonin exerts oncostatic actions and sensitizes tumor cells to chemotherapeutics or radiation. In our study, we investigated the effects of docetaxel, vinorelbine, and radiation on human breast fibroblasts and its modulation by melatonin. Docetaxel or vinorelbine inhibits proliferation and stimulates the differentiation of breast preadipocytes, by increasing C/EBPα and PPARγ expression and by downregulating tumor necrosis factor α (TNFα), interleukin 6 (IL-6), and IL-11 expression. Radiation inhibits both proliferation and differentiation through the downregulation of C/EBPα and PPARγ and by stimulating TNFα expression. In addition, docetaxel and radiation decrease aromatase activity and expression by decreasing aromatase promoter II and cyclooxygenases 1 and 2 (COX-1 and COX-2) expression. Melatonin potentiates the stimulatory effect of docetaxel and vinorelbine on differentiation and their inhibitory effects on aromatase activity and expression, by increasing the stimulatory effect on C/EBPα and PPARγ expression and the downregulation of antiadipogenic cytokines and COX expression. Melatonin also counteracts the inhibitory effect of radiation on differentiation of preadipocytes, by increasing C/EBPα and PPARγ expression and by decreasing TNFα expression. Melatonin also potentiates the inhibitory effect exerted by radiation on aromatase activity and expression by increasing the downregulation of promoter II, and COX-1 and COX-2 expression. Our findings suggest that melatonin modulates regulatory effects induced by chemotherapeutic drugs or radiation on preadipocytes, which makes it a promising adjuvant for chemotherapy and radiotherapy sensibilization.

## 1. Introduction

The growth of malignant cells is conditioned by tumor microenvironment. Normal cells that are close to the tumor cells provide them a structural support, but they are also an active component in tumor evolution. Among the different types of cells that surround malignant cells, the fibroblasts of the adipose tissue and preadipocytes are in large numbers, which are undifferentiated fibroblasts that are able to become adipocytes after adequate stimulation. The mammary adipose tissue in breast cancer not only affects the progression of the tumor but also can affect the initial stages of carcinogenesis and the response to treatment [1,2]. Fibroblasts are a main cellular element in the tumor microenvironment and epithelial–stromal interactions inhibit adipogenic differentiation and increase estrogen synthesis generating a layer of undifferentiated fibroblast with high capacity to produce estrogens around the malignant epithelial cells [1,2]. Thus, adipose cells close to tumor cells have the most aromatase expression, the enzyme that transforms androgens into estrogens, in breast tumors. Besides, the amount of adipose tissue around breast malignant cells is very large. In addition, fibroblasts close to malignant cells show the most intense aromatase immunostaining and aromatase activity and expression are 10–15 times higher than those found in malignant epithelial cells [3]. It is known that malignant epithelial cells secrete antiadipogenic cytokines, such as TNF-α, IL-11, and IL-6 that inhibit the differentiation of fibroblasts near the tumor into mature adipocytes and stimulate aromatase expression in these undifferentiated adipose fibroblasts [4]. This blockage caused by the tumor cells in adipocyte differentiation is mediated by the inhibition of expression of the two main adipogenic transcription factors CCAAT/enhancer-binding protein (C/EBP)α and peroxisome proliferator-activated receptor (PPARγ) [4]. Regulation of aromatase expression involves alternative promoter sites that support tissue-specific control. In breast cancer cells and peritumoral fibroblasts, the expression of aromatase depend on activation of promoters II and I.3 which are stimulated by prostaglandin E_2_ [3]. However, in normal breast, the low level of aromatase expression is due to the activation of promoter I.4 [5]. 

At this moment, radiation and chemotherapeutic agents play an important role in breast cancer treatment. Both therapies have positive effects on the treatment of different tumors but they have also negative side effects that repress their use since they affect the quality of life of many patients. In vivo and in vitro studies have described that irradiation exposure induces changes in adipose tissue, such as a decrease in size and number of mature adipocyte and a decrease in cell proliferation and adipogenic differentiation [6]. However, the effects of chemotherapeutic agents, such as docetaxel or vinorelbine, on adipose tissue are not known.

Melatonin, produced mainly by the pineal gland, exerts antitumor actions in different kinds of tumors but especially on hormone-dependent mammary tumors [7,8,9]. In this sense, melatonin not only decreases the situations that lead to the generation of cancer, but also exerts oncostatic actions through different mechanisms of action [9,10]. In addition, in the last years, it has been demonstrated that melatonin can sensitize tumor cells to chemotherapeutics or radiation by increasing their therapeutic effects [11,12,13,14]. In this study, we investigated the effects of ionizing radiation or chemotherapeutics, like docetaxel or vinorelbine, no longer on tumor cells but on normal cells, particularly human mammary fibroblasts, a kind of cell crucial in tumor microenvironment, and its modulation by melatonin.

## 2. Results

### 2.1. Effects of Melatonin on Docetaxel- and Vinorelbine-Induced Changes on Adipose Differentiation of Human Breast Preadipocytes

**Figure 1:**
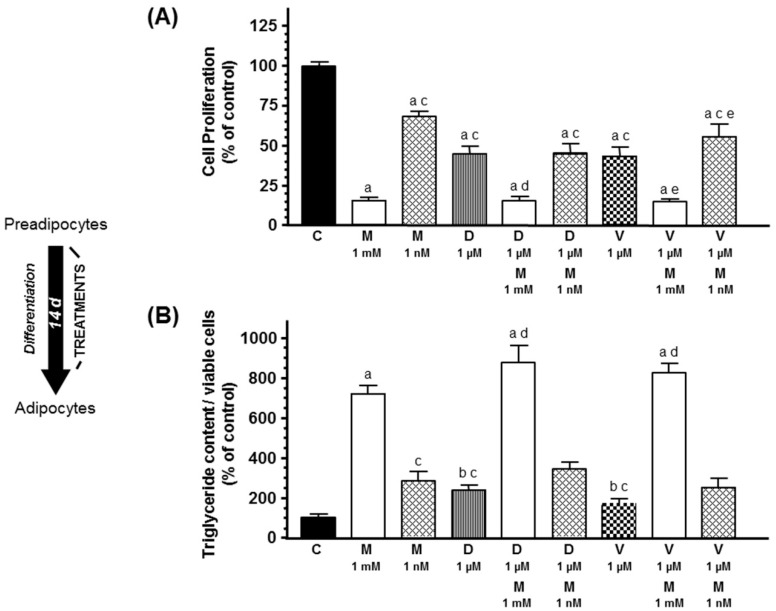
Effects of melatonin 1 mM or 1 nM on docetaxel- and vinorelbine-induced changes on (**A**) cell proliferation and (**B**) intracytoplasmic triglyceride content, during the course of differentiation of human breast adipocytes. Cell proliferation was quantified by the MTT method and triglyceride accumulation using the Oil Red O staining method. Data are expressed as the percentage of the control group (mean ± standard error of the mean (SEM)). a, *p* < 0.001 vs. C; b, *p* < 0.05 vs. C; c, *p* < 0.001 vs. M (1 mM); d, *p* < 0.001 vs. D (1 µM); e, *p* < 0.001 vs. V (1 µM). C: control, M: melatonin, D: docetaxel, V: vinorelbine.

Firstly, we investigated the effects of docetaxel and vinorelbine added to the cell cultures during the course of adipose differentiation. As shown in Figure 1, docetaxel 1 µM and vinorelbine 1 µM reduced cell proliferation and increased the triglyceride content of adipocytes, an indicator of adipogenic differentiation. Melatonin 1 mM and 1 nM treatment also decreased cell proliferation and significantly stimulated (*p* < 0.001) adipogenesis. In addition, only melatonin 1 mM potentiated the inhibitory effect on cell proliferation and the stimulatory effect of docetaxel and vinorelbine on differentiation of breast preadipocytes.

**Figure 2:**
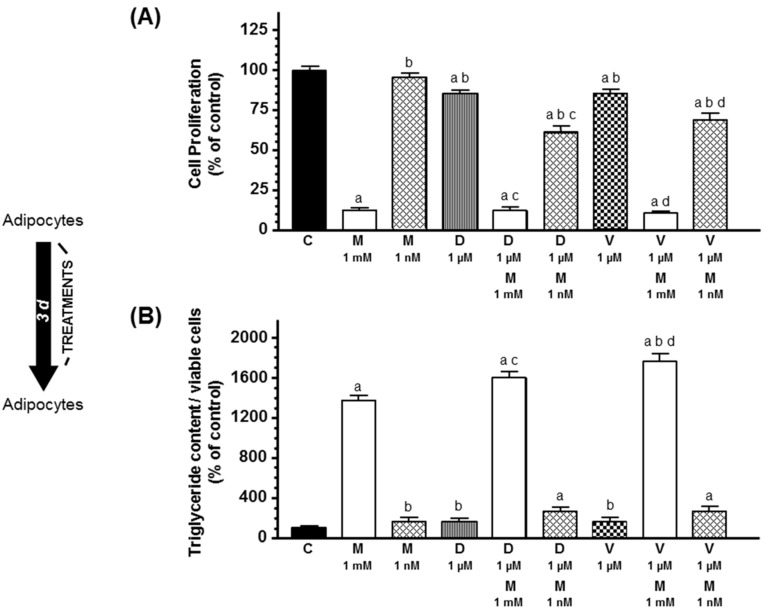
Effects of melatonin on docetaxel- and vinorelbine-induced changes on (**A**) cell proliferation and (**B**) intracytoplasmic triglyceride content on differentiating human breast adipocytes. Breast preadipocytes completely differentiated into adipocytes were treated with docetaxel (1 µM), vinorelbine (1 µM), and/or melatonin (1 mM or 1 nM) for 3 days. (**A**) Cell proliferation was measured by the MTT method. (**B**) Intracytoplasmic triglyceride accumulation was quantified by the Oil Red O staining method. Data are expressed as the percentage of the control group (mean ± SEM). a, *p* < 0.001 vs. C; b, *p* < 0.001 vs. M (1 mM); c, *p* < 0.001 vs. D (1 µM); d, *p* < 0.001 vs. V (1 µM). C: control, M: melatonin, D: docetaxel, V: vinorelbine.

Secondly, we studied the effects of docetaxel, vinorelbine, and melatonin on differentiated human breast preadipocytes. Treatment of breast adipocytes previously differentiated with docetaxel 1 µM or vinorelbine 1 µM for 3 days also induced a decrease in cell proliferation but did not modified significantly the intracytoplasmic triglyceride accumulation of the cells (Figure 2). Melatonin only at 1 mM concentration also decreased cell proliferation and increased intracytoplasmic triglyceride accumulation in combination with docetaxel or vinorelbine.

**Figure 3:**
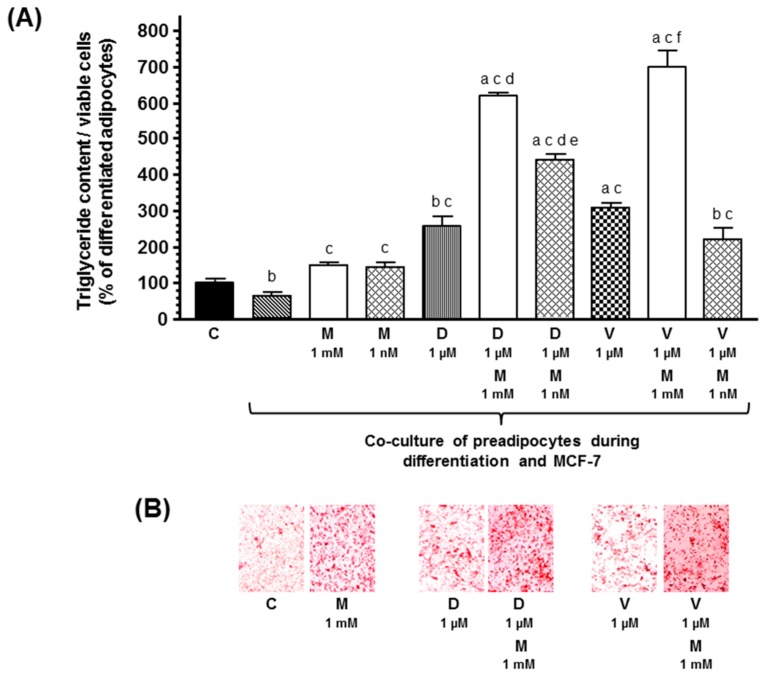
(**A**) Effects of melatonin on docetaxel- and vinorelbine-induced changes on intracytoplasmic triglyceride content in breast fibroblasts during the course of differentiation, in the presence of human breast cancer cells (MCF-7). Data are expressed as the percentage of the differentiated adipocytes (C) (mean ± SEM). a, *p* < 0.001 vs. C; b, *p* < 0.05 vs. C; c, *p* < 0.001 vs. differentiated adipocytes co-cultured with MCF-7 cells; d, *p* < 0.001 vs. differentiated adipocytes co-cultured with MCF-7 cells and docetaxel (D, 1 µM); e, *p* < 0.05 vs. differentiated adipocytes co-cultured with MCF-7 cells and D (1 µM) and melatonin (M, 1 mM); f, *p* < 0.001 vs. differentiated adipocytes co-cultured with MCF-7 cells and vinorelbine (V, 1 µM). (**B**) Representative images of the Oil Red O staining.

Then, since malignant cells secrete cytokines that prevent the differentiation of preadipocytes to adipocytes, we studied the effects of docetaxel, vinorelbine, and melatonin on adipogenic differentiation, in co-cultures of human breast preadipocytes and human breast cancer cells. According to previous findings, the presence of malignant epithelial cells decreased triglyceride content. Docetaxel and vinorelbine increased adipogenic differentiation. Melatonin only at 1 mM concentration stimulated adipogenesis differentiation and potentiated the stimulatory effect induced by docetaxel and vinorelbine (Figure 3).

### 2.2. Effects of Melatonin on Radiation-Induced Changes on Adipose Differentiation of Human Breast Preadipocytes

**Figure 4:**
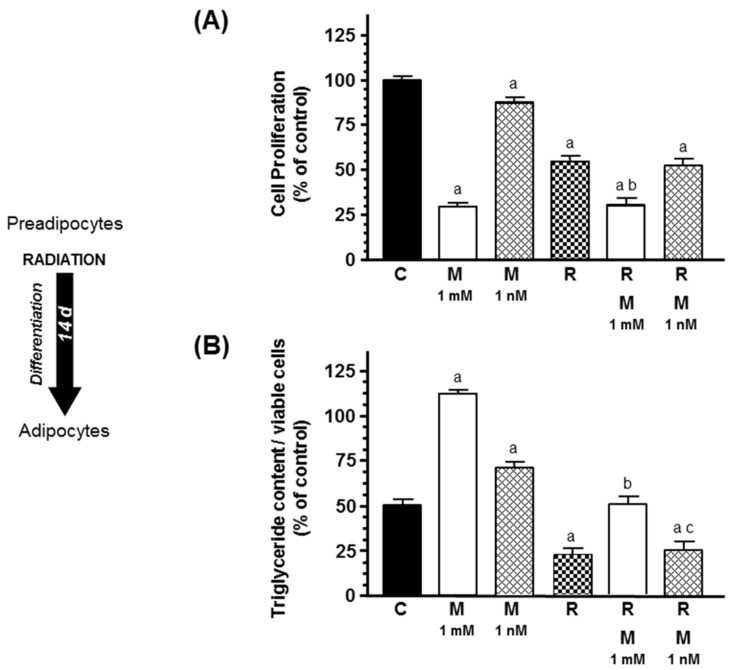
Effects of melatonin (1 mM or 1 nM) on radiation-induced changes on (**A**) cell proliferation and (**B**) intracytoplasmic triglyceride content, during the course of differentiation of human breast adipocytes. Cell proliferation was quantified by the MTT method and triglyceride accumulation using the Oil Red O staining method. Data are expressed as the percentage of the control group (mean ± SEM). a, *p* < 0.001 vs. C; b, *p* < 0.001 vs. R; c, *p* < 0.001 vs. R & M 1 mM. C: control, M: melatonin, R: radiation.

Ionizing radiation reduced significantly (*p* < 0.001) cell proliferation and intracytoplasmic triglyceride content. Melatonin (1 mM) decreased cell proliferation and counteracted the effect of radiation and increased adipogenesis differentiation (Figure 4).

**Figure 5:**
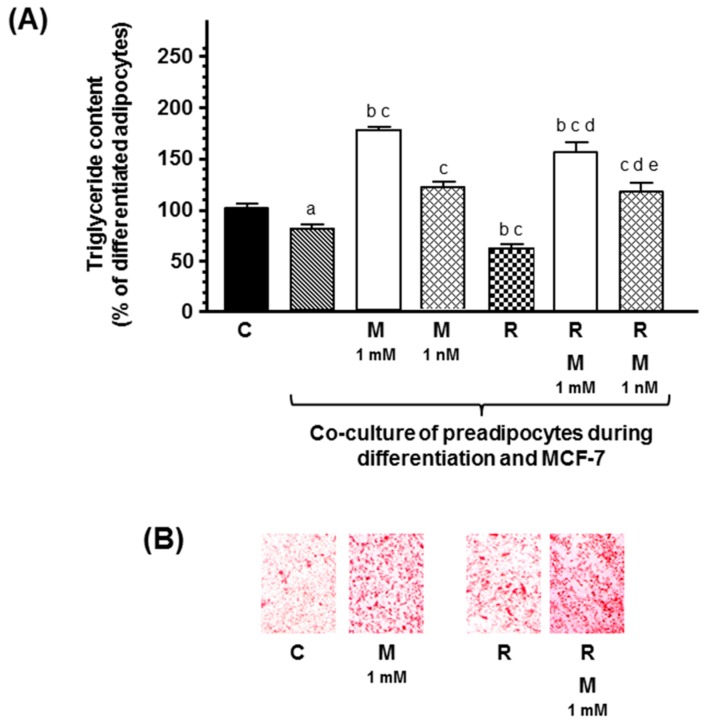
(**A**) Effects of melatonin on radiation-induced changes on intracytoplasmic triglyceride content in breast fibroblasts during the course of differentiation, in the presence of human breast cancer cells (MCF-7). Data are expressed as the percentage of the differentiated adipocytes (C) (mean ± SEM). a, *p* < 0.001 vs. C; b, *p* < 0.05 vs. C; c, *p* < 0.001 vs. differentiated adipocytes co-cultured with MCF-7 cells; d, *p* < 0.05 vs. differentiated adipocytes co-cultured with MCF-7 cells and radiated; e, *p* < 0.001 vs. differentiated adipocytes co-cultured with MCF-7 cells, radiation, and melatonin (1 mM). (**B**) Representative images of the Oil Red O staining. C: control, M: melatonin, R: radiation.

In co-cultures of human breast preadipocites and human breast cancer cells, ionizing radiation also decreased triglyceride content, and melatonin at both concentrations counteracted the effect of radiation and increased significantly adipogenesis differentiation (Figure 5). 

### 2.3. Effects of Melatonin on Docetaxel-, Vinorelbine-, and Radiation-Induced Changes on C/EBPα and PPARγ Expression in Human Breast Preadipocytes 

**Figure 6:**
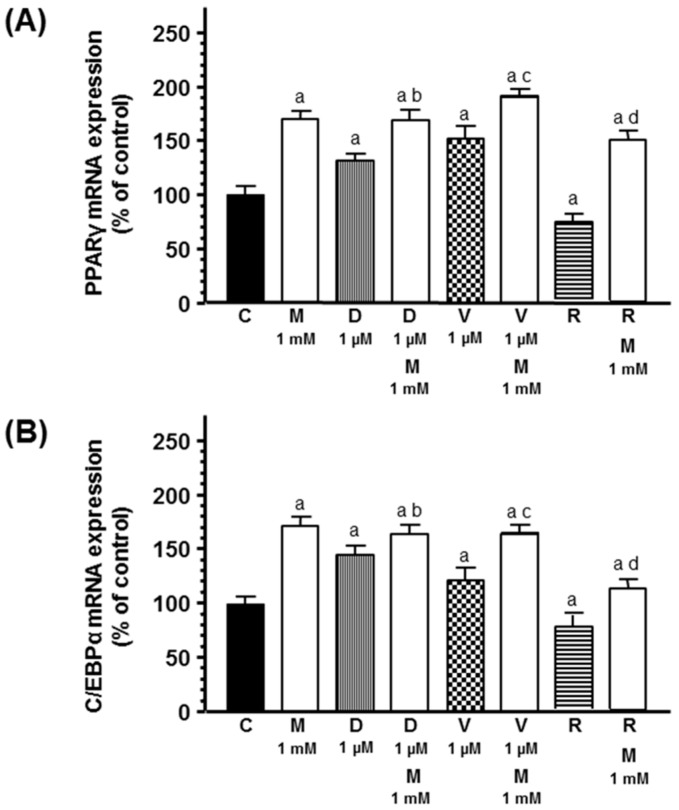
Effects of melatonin on docetaxel-, vinorelbine-, and radiation-induced changes on (**A**) PPARγ and (**B**) C/EBPα mRNA expression. Data are expressed as the percentage of control group (mean ± SEM). a, *p* < 0.01 vs. C; b, *p* < 0.05 vs. D (1 µM); c, *p* < 0.01 vs. V (1 µM); d, *p* < 0.01 vs. R. C: control, M: melatonin, D: docetaxel, V: vinorelbine, R: radiation.

Since the maintenance of the adipose phenotype depends on the expression of some specific transcription factors like C/EBPα and PPARγ, we analyzed by RT-PCR the effects of docetaxel, vinorelbine, and radiation with or without melatonin during the course of adipose differentiation on the expression of these transcription factors. Docetaxel and vinorelbine significantly increased C/EBPα and PPARγ mRNA expression. Melatonin pretreatment potentiated the stimulatory effect of chemotherapeutics on both C/EBPα and PPARγ mRNA expression. Radiation decreased C/EBPα and PPARγ mRNA expression. Melatonin counteracted the inhibitory effect exerted by radiation and significantly increased C/EBPα and PPARγ mRNA expression (Figure 6).

### 2.4. Effects of Melatonin on Docetaxel-, Vinorelbine-, and Radiation-Induced Changes on Aromatase Activity and Expression of Human Breast Preadipocytes 

**Figure 7:**
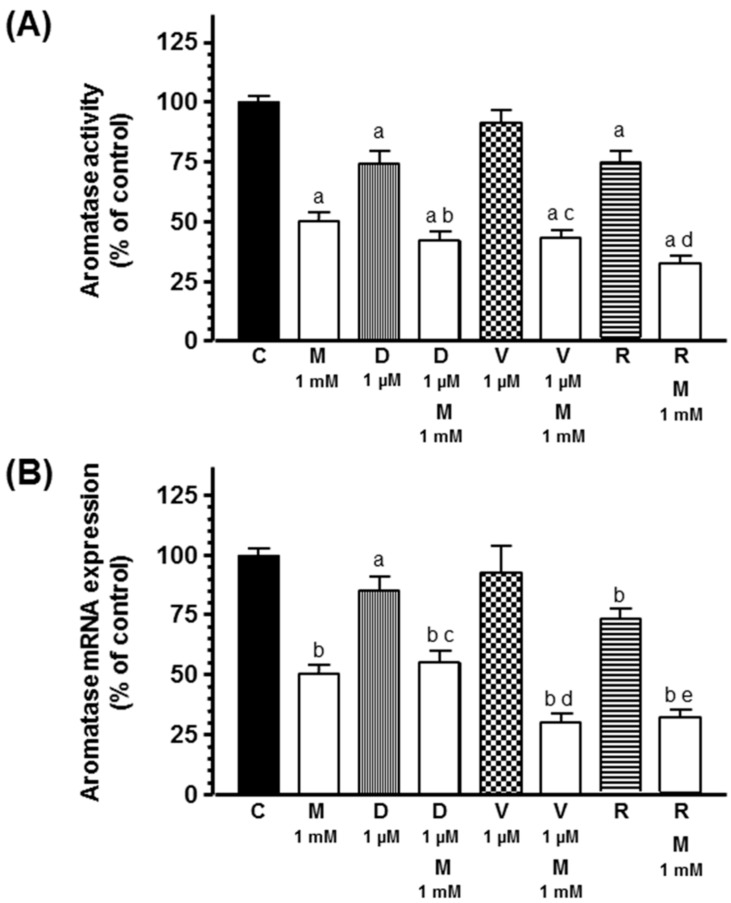
Effects of melatonin on docetaxel-, vinorelbine-, and radiation-induced changes on (**A**) aromatase activity and (**B**) aromatase mRNA expression. Data are expressed as the percentage of control group (mean ± SEM). a, *p* < 0.05 vs. C; b, *p* < 0.001 vs. C; c, *p* < 0.001 vs. D (1 µM); d, *p* < 0.01 vs. V (1 µM); e, *p* < 0.001 vs. R. C: control, M: melatonin, D: docetaxel, V: vinorelbine, R: radiation.

Since aromatase activity and expression is also considered a marked undifferentiated preadipocyte phenotype, we measured aromatase activity and expression in human breast preadipocytes and its modulation by docetaxel, vinorelbine, or radiation in the presence or not of melatonin. As shown in Figure 7, docetaxel and ionizing radiation decreased aromatase activity and expression. Melatonin inhibited aromatase activity and expression and potentiated the inhibitory effect induced by docetaxel and radiation.

**Figure 8:**
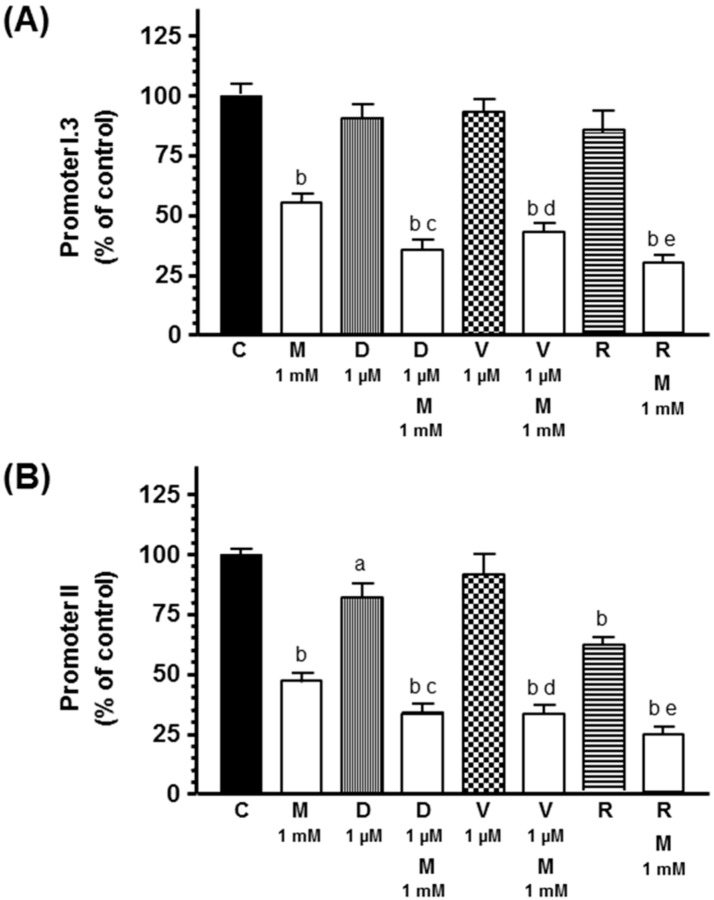
Effects of melatonin on docetaxel-, vinorelbine-, and radiation-induced changes on (**A**) aromatase promoter I.3 and (**B**) aromatase promoter II mRNA expression. Data are expressed as the percentage of control group (mean ± SEM). a, *p* < 0.05 vs. C; b, *p* < 0.001 vs. C; c, *p* < 0.001 vs. D (1 µM); d, *p* < 0.001 vs. V (1 µM); e, *p* < 0.001 vs. R. C: control, M: melatonin, D: docetaxel, V: vinorelbine, R: radiation.

With the aim of determining whether the inhibitory effect of docetaxel and radiation on aromatase activity and expression is due to the downregulation of the main aromatase promoters I.3 and II in adipocytes, we measured, by RT-PCR, the mRNA expression of both aromatase promoters. Both docetaxel and radiation decreased aromatase promoter II mRNA expression. Melatonin inhibited both aromatase promoter I.3 and II mRNA expression and potentiated the inhibitory effect induced by docetaxel and radiation on aromatase promoter II mRNA expression (Figure 8).

### 2.5. Effects of Melatonin on Docetaxel-, Vinorelbine-, and Radiation-Induced Changes on Tumor Necrosis Factor α (TNFα), Interleukin 6 (IL-6), and IL-11 mRNA Expression in Human Breast Preadipocytes 

**Figure 9:**
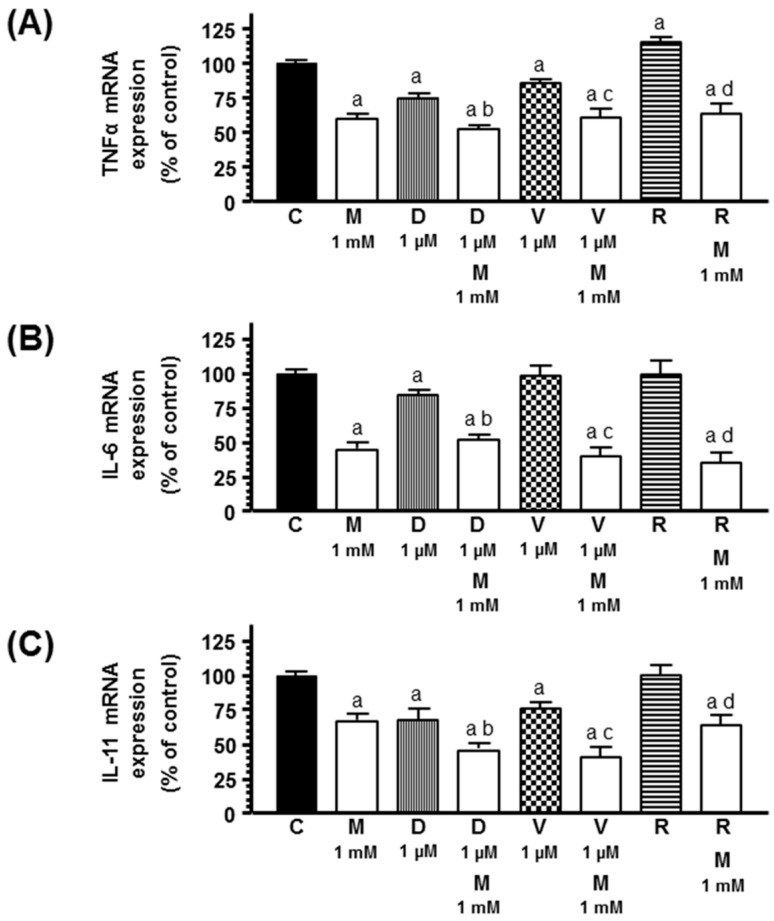
Effects of melatonin on docetaxel-, vinorelbine-, and radiation-induced changes on the expression of antiadipogenic cytokines: (**A**) tumor necrosis factor α (TNFα), (**B**) interleukin 6 (IL-6), and (**C**) IL-11. Data are expressed as the percentage of control group (mean ± SEM). a, *p* < 0.05 vs. C; b, *p* < 0.05 vs. D (1 µM); c, *p* < 0.05 vs. V (1 µM); d, *p* < 0.01 vs. R. C: control, M: melatonin, D: docetaxel, V: vinorelbine, R: radiation.

Since cytokines, such as TNFα, IL-6, and IL-11, secreted by malignant cells inhibit the differentiation of preadipocytes to adipocytes and stimulate their aromatase expression, we studied the effects of melatonin on docetaxel-, vinorelbine-, and radiation-induced changes on the expression of these cytokines. Docetaxel inhibited the expression of all three antiadipogenic cytokines. Vinorelbine reduced TNFα and IL-11 mRNA expression. Radiation increased TNFα mRNA expression. Melatonin induced a reduction of TNFα, IL-6, and IL-11 mRNA levels and significantly increased the inhibitory effect of docetaxel on these cytokines and the inhibitory effect of vinorelbine on TNFα and IL-11 mRNA expression, and counteracted the stimulatory effect of radiation on TNFα mRNA expression (Figure 9).

### 2.6. Effects of Melatonin on Docetaxel-, Vinorelbine-, and Radiation-Induced Changes on cyclooxygenase 1 (COX-1) and COX-2 mRNA Expression in Human Breast Preadipocytes 

**Figure 10:**
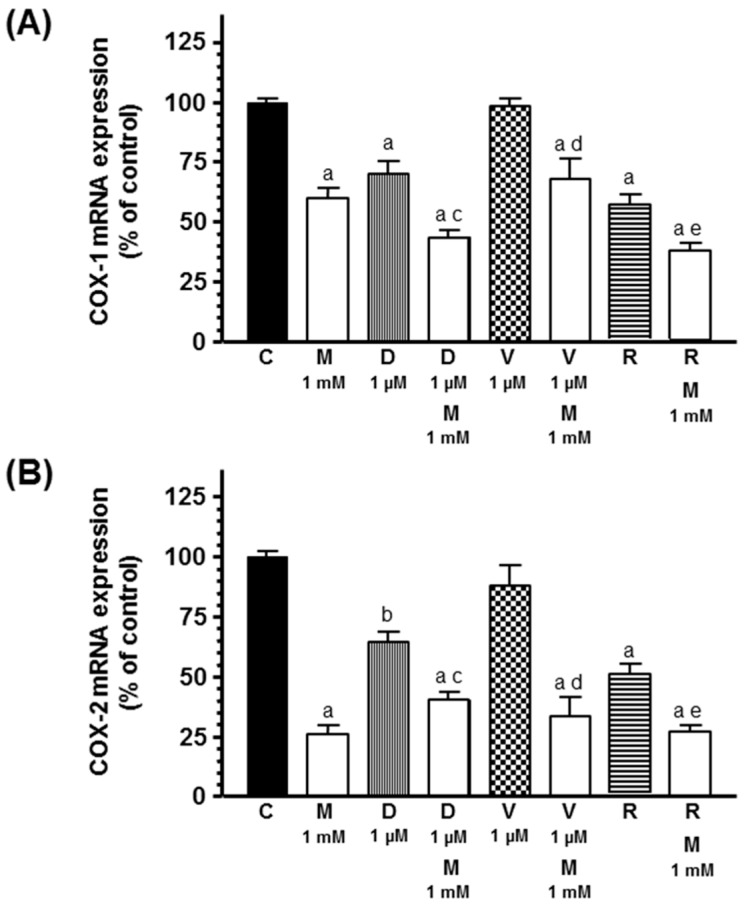
Effects of melatonin on docetaxel-, vinorelbine-, and radiation-induced changes on cyclooxygenases (COX) mRNA expression: (**A**) COX-1 and (**B**) COX-2. Data are expressed as the percentage of control group (mean ± SEM). a, *p* < 0.001 vs. C; b, *p* < 0.01 vs. C; c, *p* < 0.05 vs. D (1 µM); d, *p* < 0.001 vs. V (1 µM); e, *p* < 0.05 vs. R. C: control, M: melatonin, D: docetaxel, V: vinorelbine, R: radiation.

Since COX mRNA expression has been related with aromatase mRNA expression, we studied COX-1 and COX-2 mRNA expression in preadipocytes. Docetaxel and radiation inhibited COX-1 and COX-2 mRNA expression. Melatonin inhibited COX-1 and COX-2 mRNA expression and potentiated the inhibitory effect induced by docetaxel and radiation on COX-1 and COX-2 mRNA expression (Figure 10).

## 3. Discussion

In the formation of breast cancer, it is very important the interactions between different cells (adipocytes, preadipocytes, fibroblasts, and endothelial, adaptive, and innate immune system cells) within the tumor microenvironment and numerous signaling factors that contribute to stimulation or suppression of tumor growth [1,2]. The promotion of mammary tumors takes place inside a well-known desmoplastic reaction that involves the recruitment and accumulation of undifferentiated fibroblasts with a high aromatase activity and expression around malignant cells. Paracrine interactions between breast cancer cells and adjacent fibroblasts and endothelial cells are responsible for estrogen synthesis and lack of adipogenic differentiation in breast cancer tissue [15,16,17]. Malignant epithelial cells secrete cytokines, such as TNFα, IL-6, and IL-11, which inhibit the differentiation of the fibroblasts that surround tumor cells into adipocytes and increase their aromatase activity and expression [4,18]. Thus, tumor cells are surrounded by a large number of cells able to synthesize estrogens which promote malignant cell growth. In addition, the dynamic changes that take place within tumor microenvironment sometimes enable malignant cells to develop resistance to chemotherapeutic drugs or to radiation, because they induce a sublethal exposition of tumor cells to the different treatments [19]. Melatonin, within its antitumor actions, counteracts the effects of estrogens through different actions by interacting at different levels with estrogen signaling pathways [7,8,9,20,21,22]. Within these actions, melatonin stimulates the differentiation of preadipocytes to adipocytes and decreases aromatase activity and expression in adipose fibroblasts and cancer-associated fibroblasts, by reducing the production of estrogens near tumor cells [23,24]. Therefore, in our study, we investigated the effects of chemotherapeutics (docetaxel or vinorelbine) and ionizing radiation on the differentiation of human breast preadipocytes and its modulation by melatonin. The present study demonstrated that docetaxel, vinorelbine, and radiation have effects not only on tumor cells but also on the rest of the cells that form the tumor microenvironment. Docetaxel and vinorelbine inhibited cell proliferation and increased the triglyceride content of adipocytes, an indicator of adipogenic differentiation, both in breast preadipocytes cultures and in co-cultures of breast preadipocytes with human breast cancer cells. Since it is known that malignant cells secrete antiadipogenic cytokines that prevent the differentiation of preadipocytes to adipocytes near the tumor, being this inhibition of differentiation the main mechanism responsible for desmoplastic reaction, in some experiments, we used co-cultures of breast preadipocytes and breast cancer cells. Melatonin 1 mM potentiated this stimulatory effect on differentiation of preadipocytes induced by docetaxel and vinorelbine. Until now, there are several studies about melatonin and adipogenesis but with contradictory results. Thus, some authors [23,25,26] described a dose-dependent stimulatory action of melatonin on adipocyte differentiation in 3T3-L1 fibroblasts by upregulating C/EBPα and PPARγ. Recently, Yang et al. (2017) [27] described that melatonin promotes the differentiation of bovine intramuscular adipocytes into adipocytes by increasing C/EBPα, C/EBPβ, and PPARγ mRNA expression via MT2 melatonin receptor. However, some studies point to the fact that melatonin inhibits adipogenic differentiation through a downregulation of C/EBPα, C/EBPβ, and PPARγ [28]. Thus, the implication of melatonin in adipogenesis is not completely clear and the melatonin actions might be different depending on animal species or the melatonin doses employed. Raloxifene, a selective estrogen receptor modulator like melatonin, also stimulates adipocyte differentiation of 3T3-L1 cells [29]. The conversion of preadipocytes into mature adipocytes depends on a cascade of activation of transcription factors that modulates the expression of different proteins involved in the establishment of the mature fat cell phenotype. In this sequence of activation, two of the main regulators of terminal adipogenesis are PPARγ and C/EBPα, which induce the transcription of several adipocyte genes encoding proteins and enzymes involved in promoting and maintaining the adipocyte phenotype [29,30]. Docetaxel and vinorelbine significantly increased C/EBPα and PPARγ mRNA expression. Melatonin treatment potentiated the stimulatory effect of both chemotherapeutics on C/EBPα and PPARγ mRNA expression. The effects of melatonin on normal and cancer cells biology depend on different factors like concentration, time of exposure, or specific characteristics of cells. Previously, it has been described that melatonin exerts an inhibitory effect on human breast cancer cells proliferation only at nanomolar concentration [9,18,20]. However, high melatonin doses are required to obtain oncostatic effects in other types of normal and cancer cells, like human prostate cancer cell, neuroblastoma, human Ewing sarcoma, colon cancer cells, hepatocarcinoma cells, fibroblasts, or endothelial cells [18,23,31,32]. Melatonin crosses the blood–brain barrier because it is a highly lipid-soluble indolamine and its concentration in the cerebrospinal fluid is higher than in blood. It is known that melatonin is also 3 orders of magnitude more concentrated in neoplastic and adipose tissue of the breast [33]. These high levels of melatonin in some tissues may contribute to explaining why high concentrations of melatonin are necessary to obtain some oncostatic actions of melatonin. 

On the other hand, in our study, radiation reduced both cell proliferation and intracytoplasmic triglyceride content. Melatonin 1 mM decreased cell proliferation and counteracted the effect of radiation and increased adipogenic differentiation. The inhibitory effect of radiation on adipogenic differentiation was also mediated by the regulation of C/EBPα and PPARγ. Thus, radiation decreased C/EBPα and PPARγ mRNA expression. Melatonin counteracted the inhibitory effect exerted by radiation and significantly increased C/EBPα and PPARγ mRNA expression. Our data are consistent with previous observations that demonstrated that irradiation inhibits the osteogenic and adipogenic ability of bone marrow mesenchymal stem cells [34]. Ultraviolet A also inhibits adipogenic differentiation of human adipose tissue-derived mesenchymal stem cells by reducing C/EBPα and PPARγ mRNA expression [35]. In a recent study, Shreder et al. (2018) [36] described that high X-ray doses induce a dose-dependent decrease of the proliferation and an increase in lipid accumulation in human preadipocytes. They described that the expression of markers of adipogenic differentiation (C/EBPα, PPARγ, and C/EBPβ) is not significantly changed upon irradiation and they suggested a radiation-induced response relates to inflammation. The stimulatory effects of melatonin on C/EBPα and PPARγ mRNA expression have already been described in 3T3-L1 fibroblasts through known melatonin receptor-mediated mechanisms [23].

Another marked undifferentiated preadipocyte phenotype is the aromatase expression [15,37]. Docetaxel and radiation decrease aromatase activity and expression and this effect was significantly potentiated by melatonin treatment. At this moment, the effects of ionizing radiation on the local estrogen biosynthesis are not well known. In the case of docetaxel, it has been described that it decreases intratumoral aromatase mRNA levels in breast tumors [38]. Our results are in agreement with those describing that melatonin decreases aromatase activity and expression in human breast cancer, glioma, and endothelial cells as well as in 3T3-L1 fibroblasts or breast cancer-associated fibroblasts [22,23,24,39,40]. Breast adipose fibroblasts are the major source of local estrogen biosynthesis and maintain low levels of aromatase expression via promoter I.4. However, in breast tumor fibroblasts and breast malignant epithelial cells, aromatase expression is induced via stimulation of promoters II and I.3 [3]. In our study, the inhibition of aromatase mRNA expression induced by docetaxel and vinorelbine can be explained through a downregulation of the promoter II mRNA expression. Melatonin inhibited promoter II and I.3 mRNA expression and potentiated the inhibitory effect induced by docetaxel and radiation on promoter II. It is known that melatonin downregulates aromatase mRNA expression and the gene expression of aromatase promoter II, I.3 and I.4 in human breast cancer cells and in breast adipose fibroblasts [23,24]. The increases of aromatase promoters II and I.3 in breast adipose fibroblasts are also related with the increase in cellular cAMP levels through PGE_2_ secreted by malignant epithelial cells [41]. A relationship between aromatase and the expression of COX enzymes has been proposed, in a way that an increased COX activity elevates PGE_2_ levels, which increase intracellular cAMP that activate promoters II and I.3 and result in an increased aromatase expression. COX-1 and COX-2 are rate-limiting enzymes that catalyze the conversion of arachidonic acid to prostaglandins. COX-1 is constitutively expressed in most cells and tissues and COX-2 is undetectable but inducible in inflammation or cancer [42,43,44]. High levels of PGE_2_ have been associated with stimulation of angiogenesis and cancer development [45]. In our study, docetaxel and radiation decreased both COX-1 and COX-2 mRNA expression and melatonin inhibited the expression of both COX and potentiated the inhibitory effect of docetaxel and radiation. This inhibition of COX expression could explain the reduction of promoter II mRNA expression and the inhibition of aromatase expression exerted by docetaxel and radiation. Docetaxel also inhibits COX-2 induction in vascular smooth muscle cells [46]. However, docetaxel induces COX-2 and increases PGE_2_ synthesis in tumor cells, which is a disadvantage in anticancer therapy with docetaxel, since this fact affects tumor development and metastasis [47]. In human breast cancer cells, one of the mechanisms through melatonin modulates aromatase enzyme is also via a downregulation of COX-1 and COX-2 mRNA expression [48]. Then, we can suggest that, in our study, the downregulation of COX mRNA expression could decrease the production of PGE_2_ by preadipocytes and the lower levels of PGE_2_ diminish the intracellular levels of cAMP and consequently the activation of aromatase promoter II that result in decreased aromatase expression. 

In addition, as we previously described, breast cancer cells secrete antiadipogenic cytokines that inhibit the differentiation of preadipocytes to adipocytes and increase their aromatase activity and expression [3,4,49,50,51]. It has been described that melatonin, in co-cultures of human breast cancer cells and 3T3-L1 fibroblasts, decreases the levels of TNFα, IL-11, and IL-6 in the culture media and downregulates TNFα, IL-11, and IL-6 mRNA expression in both types of cells [52]. We found that docetaxel decreased TNFα, IL-11, and IL-6 expression and vinorelbine reduced TNFα and IL-11 expression. These results agree with previous descriptions in which docetaxel downregulated intratumoral aromatase mRNA expression through an inhibition of intratumoral TNFα mRNA expression in human breast cancer [39]. In addition, Tsavaris et al. (2002) [53] described a decrease in TNFα levels in breast cancer patients after treatment with taxanes. The reduction of TNFα, IL-11, and IL-6 expression induced by the chemotherapeutics may also contribute toward explaining the stimulation of differentiation exerted by them. Melatonin increased the inhibitory effect exerted by docetaxel and vinorelbine on these antiadipogenic cytokines, which explains that melatonin potentiated the stimulatory effect exerted by docetaxel and vinorelbine on differentiation of preadipocytes. However, radiation increased TNFα mRNA expression which justifies why radiation inhibits adipogenic differentiation. The increase of TNFα expression after irradiation has been also described in human adipose during radiotherapy [54]. Melatonin counteracted this radiation-induced effect and decreased TNFα mRNA expression stimulating adipogenic differentiation.

In summary, the results of our study indicated that chemotherapeutics, like docetaxel or vinorelbine, may play a role in the desmoplastic reaction in breast cancer. They inhibited proliferation and stimulated the differentiation of human breast preadipocytes, by increasing the expression of the two main regulators of terminal adipogenesis, C/EBPα and PPARγ, and by downregulating antiadipogenic cytokines, like TNFα, IL-6, or IL-11. Radiation inhibited both proliferation and differentiation of preadipocytes to adipocytes through the downregulation of C/EBPα and PPARγ and by stimulating TNFα. In addition, docetaxel and radiation decreased aromatase activity and expression by decreasing aromatase promoter II mRNA expression and COX-1 and COX-2 expression. Melatonin potentiated the stimulatory effect of docetaxel and vinorelbine on differentiation and its inhibitory effect on aromatase activity and expression, by increasing the stimulatory effect on C/EBPα and PPARγ and by increasing the downregulation of antiadipogenic cytokines and COX expression. In combination with radiation, melatonin counteracted the inhibitory effect of radiation on differentiation of preadipocytes, by increasing C/EBPα and PPARγ expression and by decreasing the TNFα expression induced by radiation. Melatonin also potentiated the inhibitory effect exerted by radiation on aromatase activity and expression by increasing the downregulation of promoter II, and COX-1 and COX-2 expression. Within tumor microenvironment, melatonin modulates the response of preadipocytes (differentiation, aromatase activity, and expression) to chemotherapeutic drugs or to radiation. Recently, some studies [2,55,56] suggest melatonin as an adjuvant for chemotherapy and radiotherapy sensibilization showing synergistic antitumoral effects.

## 4. Materials and Methods 

### 4.1. Cells and Culture Conditions

Human breast preadipocytes were purchased from ZenBio, Inc (Research Triangle Park, NC, USA). They were maintained as monolayer cultures in 58,2 cm^2^ plastic culture plates in Preadipocyte Medium (PM-1) (ZenBio, Inc, Research Triangle Park NC, USA) supplemented with penicillin (20 units/mL) and streptomycin (20 µg/mL) (Sigma-Aldrich, Madrid, Spain) at 37 °C in a humid atmosphere containing 5% CO_2_. Cells were cultured until reaching confluence and then they were differentiated into the adipocyte phenotype with Adipocyte Differentiation Medium (DM-2) (ZenBio, Inc, Research Triangle Park NC, USA) for 14 days. Medium change was realized every 2 days. To determine the effects during the course of adipocyte differentiation, docetaxel (1 µM), vinorelbine (1 µM), and/or melatonin (1 mM or 1 nM), or vehicle were maintained in the cultures during the fourteen-day period. To study the effects on differentiation of human breast preadipocyte cells, docetaxel 1 µM, vinorelbine 1 µM, and/or melatonin 1 mM or 1 nM) or vehicle were added to the cultures at day 14 of the differentiation period and maintained for 72 h. In the other experiments, cells were cultured for 24 h in Preadipocyte Medium (PM-1), irradiated at 8 Gy and differentiated into the adipocyte phenotype with Adipocyte Differentiation Medium (DM-2) in the presence or not of melatonin (1 mM or 1 nM) for 14 days. To study the effects on differentiation of human breast preadipocyte cells, they were irradiated at day 14 of the differentiation period at 8 Gy and incubated in the presence or not of melatonin (1 mM or 1 nM) for 72 h.

### 4.2. Co-culture of Human Breast Preadipocites and MCF-7 Cells

In some experiments, we employed co-cultures of human breast preadipocytes and human breast cancer cells (MCF-7). Based on previous works [52], cells were co-cultured using Falcon 24-multiwell plates and Falcon cell culture inserts. Human breast preadipocytes were plated (90 × 10^3^ cells/well) on the bottom wells in Preadipocyte Medium (PM-1) and incubated overnight. At the same time, MCF-7 cells (5 × 10^3^ cells) were seeded on the permeable membrane (0.45 µm) of the tissue-culture inserts in DMEM supplemented with 10% FBS also for 24 h. Human breast preadipocytes and MCF-7 cells were cultured separately for 24 h to establish attachment. After 24 h, MCF-7-seeded inserts were moved over human breast preadipocytes cultures in the 6-well plates to create the hanging co-culture setup and the medium was changed to the Adipocyte Differentiation Medium (DM-2) with docetaxel (1 µM), vinorelbine (1 µM), and/or melatonin (1 mM or 1 nM), or vehicle. In some experiments, cells were irradiated at 8 Gy and cultured in the presence or not of melatonin (1 mM or 1 nM) or vehicle. Due to the membrane pore size and diffusional distance between cells within this setup, cell-to-cell contact was prevented but paracrine signalling could occur between endothelial cells in the 24-well plate and epithelial cells on the insert. After 14 days, human breast preadipocyte differentiated in the bottom plate were evaluated for differentiation to mature adipocytes (quantification of triglycerides by Oil Red O staining method), for proliferative indices (MTT method) or for aromatase activity.

### 4.3. Ionizing Radiation Treatment

Human breast preadipocytes and MCF-7 cells were exposed to X irradiation using a model YXLON SMART 200 tube (Yxlon International, Hamburg, Germany) at room temperature. We used 8 Gy radiation as the optimal radiation dose, as previously described [11].

### 4.4. Measurement of Cellular Proliferation 

Cell proliferation was measured by the MTT [3(4,5dimethylthiazol-2-yl)-2,5-diphenyl tetrazolium bromide] method, since the reduction of tetrazolium salts is broadly accepted as an accurate way to study cell proliferation [57]. At the end of the experiments, cell proliferation was measured reading the absorbance at 570 nm in a microplate reader (Labsystems Multiskan RC 351, Vienna, VA, USA). MTT was obtained from Molecular Probes Inc. (Eugene, OR, USA). 

### 4.5. Quantitation of Triglycerides by Oil Red O Staining

We used the Oil Red O staining method to quantify the accumulation of intracytoplasmic triglyceride [15]. As previously described [52], cells were fixed in 10% *p*-formaldehyde, washed, and stained with oil red before photographing (20×). To determine the content of lipids, a colorimetric method was used. 

### 4.6. Measurement of Cellular Aromatase Activity

Aromatase activity in human breast preadipocytes cells was measured by the tritiated water release assay, based on the formation of tritiated water during aromatization of a labeled androgenic substrate [1β-^3^H(N)]-androst-4-ene-3,17-dione [58]. At the end of the experiments, human breast preadipocytes differentiated into mature adipocytes in the presence of docetaxel (1 µM), vinorelbine (1 µM), and/or melatonin (1 mM or 1 nM), or vehicle during the course of adipocyte differentiation or these human breast preadipocytes completely differentiated into adipocytes and exposed for 72 h to docetaxel (1 µM), vinorelbine (1 µM), and/or melatonin (1mM or 1 nM), or vehicle (ethanol at a final concentration lower than 0.0001%) were cultured with serum-free media containing 100 nM [1β-^3^H(N)]-androst-4-ene-3,17-dione] (NEN Life Science Products, Boston, MA, USA) (25–30 Ci/mM) in the presence of chemotherapeutics and/or melatonin or irradiated. As previously described [10], successive extractions with organic solvents were performed to separate the steroids from the aqueous phase.

### 4.7. Measurement of Specific mRNA Gene Expression

At the end of the experiments, analyses of different genes mRNA expression in human breast preadipocytes were realized by real-time RT-PCR. The total cellular RNA was purified with the Nucleospin RNA II Kit (Macherey-Nagel, Düren, Germany) following the manufacturer´s instructions. Integrity of RNA was assessed by electrophoresis in ethidium bromide-stained 1% agarose-Tris-borate EDTA gels. The absorbance ratio of A_260nm_/A_280nm_ was greater than 1.8. For cDNA synthesis, 0.5 μg of total RNA was denaturated at 65 °C for 10 min and reverse transcribed for 50 min at 45 °C with cDNA Synthesis kit (Bioline, London, UK) in a final volume of 20 μL in the presence of 500 ng of oligo (dT) 12–18 primer. The primers used for amplification (Sigma Genosys Ltd., Cambridge, UK), using the housekeeping gene *S14* as a control quantification, are listed in Table 1. RT-PCRs were performed in a MX3005P system (Stratagene, La Jolla, CA, USA) using Brilliant ® SYBR ® Green PCR Master Mix (Applied Biosystems, Madrid, Spain) following the manufacturer’s instructions. Amplifications were performed for 40 cycles using the following temperature profile: 60 °C, 45 s (annealing); 72 °C, 30 s (extension); and 95 °C, 30 s (denaturation). Each reaction was run ninefold by quadruplicate. Melting curves were performed to verify that only a single product with no primer-dimers was amplified. For the primers used, there were no differences between transcription efficiencies, and the fold-change in each sample was calculated by the 2^–∆∆^Ct method [59]. The fractional cycle at which the amount of amplified targets became significant (Ct) was automatically calculated by the PCR program. 

### 4.8. Statistical Analysis

Statistical analyses were realized using GraphPad Prism software. The data are expressed as the mean ± the standard error of the mean (SEM). Differences between groups were analyzed by using one way analysis of variance (ANOVA), followed by the Student–Newman–Keuls test. Results were considered as statistically significant at *p* < 0.05.

## Figures and Tables

**Table 1:** Primers used for amplification of mRNA transcripts.

mRNA	Sequence	Concentration (nM)
hARO fw	5′- GTCGTGGACTTGGTCATGC -3′	100
hARO rv	5′- CGAGTCTGTGCATCCTTCC -3′	100
hC/EBPα fw	5′- AGGTGCTGGAGCTGACCAGT -3′	200
hC/EBPα rv	5′- AAGCCTCGAGATCCGGCGAC -3′	200
COX-1 fw	5′- ACCCGCACGGGCTATTCCGGC -3′	200
COX-1 rv	5′- AGGCGCATGAGCATCTCTCGG -3′	200
COX-2 fw	5′- ATGTATGAGTGTGGGATTTGA -3′	200
COX-2 rv	5′- TCCAAAATCCCTTGAAGTGGG- 3′	200
hIL-6 fw	5′- AGGAGACTTGCCTGGTGAAA -3′	200
hIL-6 rv	5′- CAGGGGTGGTTATTGCATCT -3′	200
hIL-11 fw	5′- GCTGGTTTCGAACTCCTGAC -3′	200
hIL-11 rv	5′- CAGGGTGACTTGTGGAACCT -3′	200
hpII fw	5′- CTCTGAAGCAACAGGAGCTATAGA -3′	100
hpII rv	5′- CAGGCACGATGCTGGTGATG -3′	100
hpI.3 fw	5′- GGGCTTCCTTGTTTTGACTGTAA -3′	200
hpI.3 rv	5′- AGAGGGGGCAATTTAGAGTCTGTT -3′	200
hPPARγ fw	5′- GATGCACTGCCTATGAGCACTT -3′	400
hPPARγ rv	5′- AGAGGTCCACAGAGCTGATTCC -3′	400
hS14 fw	5′- TCCTGCGAGTGCTGTCAGAG -3′	100
hS14 rv	5′- TCACCGCCCTACACATCAAAC -3′	100
hTNFα fw	5′- TGGGGTTTGTGAAACTGTGA -3′	50
hTNFα rv	5′- GTTCCTGCACATTCCCTCTC -3′	50
